# Collagen scaffold for mesencyhmal stem cell from stromal vascular fraction (biocompatibility and attachment study): Experimental paper

**DOI:** 10.1016/j.amsu.2020.07.055

**Published:** 2020-08-18

**Authors:** Panji Sananta, I.G.M.O. Rahaditya, Mohamad I. Imadudin, Marvin A. Putera, Sri Andarini, Umi Kalsum, Edi Mustamsir, Respati S. Dradjat

**Affiliations:** a Doctoral Program of Medical Science, Faculty of Medicine, Universitas Brawijaya, Indonesia; b Orthopaedic and Traumatology Department, Faculty of Medicine, Universitas Brawijaya, Indonesia; c Public Health Department, Faculty of Medicine, Universitas Brawijaya, Indonesia; d Pharmacology Department, Faculty of Medicine, Universitas Brawijaya, Indonesia

**Keywords:** Collagen scaffold, Mesenchymal stem cell, Stromal vascular fraction

## Abstract

**Background:**

One of the most important part of tissue engineering (TE) is a matrix called scaffold. A good scaffold integrates with the host tissue and support the growth and differentiation of the cells. Collagen is the most abundant protein in the ECM and has been considered to be a group of proteins with a characteristic molecular structure—fibrillar structure, which contributes to the extracellular scaffolding.

**Objective:**

In this research we study the biocompatibility and attachment of collagen scaffold by measuring the level of availability of mesenchymal stem cell (MSC) cluster from stromal vascular fraction (SVF).

**Method:**

This study was experimental invitro on MSC culture derived from SVF, with post-test control group design. Biocompatibility was measured by viability of MSC from SVF with marker Propidium Iodine through flowcytometry and electron microscope was used to assess the population density of MSC from SVF by measuring the number of cluster cells seen.

**Result:**

Oxidize cellulose has the greatest value of MSC cluster with average number of 2003 cell cluster. This result was significant with p < 0.05 using One-Way Anova and Tukey Test.

**Conclusion:**

Collagen scaffold is ideal for MSC from SVF because of its compatibility and attachment.

## Highlights

• Biocompatibility and attachment of collagen scaffold.
• Measuring the level of availability of mesenchymal stem cell (MSC) cluster from stromal vascular fraction (SVF).
• Experimental in vitro study on MSC culture derived from SVF.
• Biocompatibility was measured by viability of MSC from SVF with marker Propidium Iodine through flowcytometry.
• Electron microscope was used to assess the population density of MSC from SVF.

## 1 Introduction

Tissue engineering is a multidisciplinary science that applies the principles of bioengineering for the fabrication of new and improved biomaterials capable of maintaining and restoring the functionality of organs and tissues impaired by disease and trauma. This translational approach has been applied to develop and design patient-specific tissue grafts that mimic the functional properties of native tissues. Three important factors have been accredited to the success of tissue engineering: cocultured stem cells, signaling factor, and the bio-fabricated scaffold [1].

Fundamentally, the tissue engineering paradigm consists of scaffolds, signals, and cells, These 3 elements can be combined or used independently to attempt to generate tissues in a limitless number of arrangements. However, with an increasing complexity of design, there are greater challenges to translation. For example, receiving regulatory approval for an acellular scaffold requires substantially less time and fewer resources than does a drug-eluting scaffold that has been pre-seeded with stem cells [1].

The stem cells are capable of differentiating into several types of tissues and organs, while the bio-fabricated scaffold provides structural support to the seeded stem cells. Signaling factors are responsible for influencing cell phenotype, metabolism, migration, and organization. Stem cells are undifferentiated cells of embryonic, fetal origin, and they possess the ability to give rise to differentiated cells and then finally develop into organs. Stem cell characteristics include the ability to self-replicate and renew, clonage forming, and high potency ability. In terms of the potency ability of stem cells, stem cells can be totipotent, could differentiate into any cell types (pluripotent), and could differentiate into cells that arise from the three germ layers—ectoderm, endoderm, and mesoderm—from which organs develop. Stem cells can be categorized broadly into embryonic and adult stem cells and are efficient cell sources for tissue regenerative applications. They have also been reported to have the abilities to promote tissue homeostasis, growth, and repair, thereby contributing importantly to tissue and organ regeneration. Bio-fabricated scaffolds consist of decellularized biomaterials to provide structural and anatomical functions to the seeded stem cells, thereby resulting into successful formation of specific tissue [2].

MSCs are stromal stem cells that are heterogeneous and are derived from several tissue sources that include adipose tissue, periodontal ligaments, bone marrow, umbilical, placenta, and lungs. MSCs express surface markers like CD73, CD44, CD90, and CD105. The most widely known and used MSCs are bone marrow MSCs and adipose tissue-derived MSCs isolated and purified from the bone marrow and adipose tissue, respectively [3].

Stem cells have self-renewal and multipotency abilities that may help in creating advanced tissue engineered skin substitutes. Among the main sources of cells that may be used to engineer such substitutes are adult stem cells, embryonic stem cells (ESCs), and induced pluripotent stem cells (iPSCs). These cells have the potential to secret paracrine factors, making them an attractive option for the treatment of acute and chronic wounds. So far, adult stem cells, especially mesenchymal stem cells (MSCs), have been widely used for regenerative healing. MSCs can self-renew and have the ability to differentiate into osteoblastic, adipocytic, and chondrocytic lineages [4].

Collagen is the most abundant protein in the ECM and has been considered to be a group of proteins with a characteristic molecular structure—fibrillar structure, which contributes to the extracellular scaffolding. That is to say, collagen plays an important role in maintaining the biological and structural integrity of ECM and provides physical support to tissues. Collagen possesses extensive sources (such as bone, cartilage, tendon, ligament, blood vessel, nerve, skin), as it is the main structural protein of most hard and soft tissues. In addition, collagen offers low immunogenicity, a porous structure, permeability, good biocompatibility and biodegradability and has functions to regulate the morphology, adhesion, migration and differentiation of cells. All of these good performances make this natural polymer seem to be a promising biomaterial for scaffolds in tissue engineering. However, the collagen scaffolds lack mechanical strength and structural stability upon hydration, which limit their applications in particular tissues. Intermolecular cross-linking of collagen scaffolds can be achieved by physical or chemical methods, which can improve the mechanical properties of the scaffold. Besides, blending collagen with other materials, such as natural, synthetic polymers and inorganic materials, is also frequently used to enhance the mechanical strength of collagen scaffolds. Meanwhile, biochemical factors could be added into or modified onto the scaffold selectively according to the damaged region to improve the cellular outcome [5].

In this study we want to know biocompatibility and attachment of collagen scaffold for mesenchymal stem cell derived from stromal vascular fraction.

## 2 Methods

This study was experimental invitro on MSC culture derived from SVF, with post-test control group design. This study was conducted at Biomedic Laboratory of Faculty of Medicine Universitas Brawijaya Malang.

Collagen scaffold was obtained from commercially use biomaterial with the brand Lyostipt

Oxidize cellulose obtained from commercially use biomaterial with the brand surgicell

Stromal Vascular Fraction was originated from Rattus novergicus fat tissue with ZUK method [6].

Biocompatibility was measured by viability of MSC from SVF with marker Propidium Iodine through flowcytometry.

Electron microscope: Assessment of population density of MSC from SVF by measuring the number of cluster cells seen in imaging scanning electron microscope/SEM to reduce measurement bias, then measured the number of cluster cells by two observers, namely researchers and expert analysts in the field of stem cells. In addition, cells measured for density on SEM imaging were performed under a light microscope to prove that cells imaged in SEM were adipocyte derived stem cells.

## 3 Result

From Table 1 and Table 2 we know that oxidize cellulose has viability only 19.528, collagen 93.273 and control 95.995. Since the data is not homogen we proceed to parametric analysis, kruskal wallis test and Mann Whitney test.

**Table 1:** Chi-Square test result.

	N	Mean	Std. Deviation
Control	4	95.995	0.428
Group 2 (Oxidize Cellulose) l	4	19.528	2.543
Group 3 (Collagen) l	4	93.273	1.195

**Table 2:** Viability test.

Chi-Square	Df	p	Result
17.99	5	0.003	Significant

### 3.1 Kruskal wallis examination

Based on the results of the Kruskal Wallis analysis, found that the p value for the Cell Number is 0.003. Because for the parameter Cell Number has a value of p < 0.05, which means that there is a significant difference in effect between treatments at an error rate of 5%

### 3.2 Mann whitney examination

Mann Whitney test results in Table 3 showed that the control group gave significant differences with all treatment groups. Likewise, the treatment group for Oxidized Cellulose has a significant difference with all treatments and controls.

**Table 3:** Mann-Whitney test.

Group comparison		Sig.	Result
Control	Oxidize Cellulose	0.021	Significant
	Collagen	0.021	Significant
Oxidize Cellulose	Collagen	0.021	Significant

**Table 4:** Number of cluster analysis.

	N	Mean	Minimum	Maximum
Control	4	170.750	159	186
Oxidize cellulose	4	2003.000	1866	2086
Collagen	4	918.250	842	985

a. The value of the control observations obtained an average value of 170.750 shown by Table 4.

b. The observed values of oxidize cellulose obtained an average value of 2003,000.

c. Collagen observations from obtained an average value of 918,250.

**Fig. 1:**
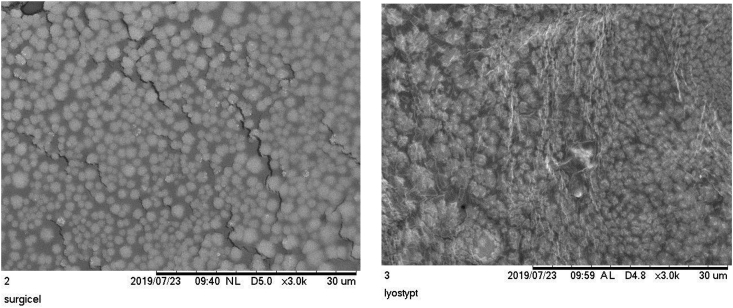
Electrone microscope Scanning from mesenchymal stem cell to collagen with 3000x magnification.

These are an electron microscope examination of the MSC attachment to scaffold showed in Fig. 1.

Electrone microscope Scanning from mesenchymal stem cell to oxidize cellulose with 3000x magnification.

Since the data homogen and normally distributed, we used parametric study, One way Annova and Tukey Test.

### 3.3 One way Annova Examination

Based on the ANOVA analysis results it is found that the p value is 0,000 which means that there is a significant difference in effect between treatments at an error rate of 5%.

Tukey's test results in Table 5 showed that the control group with the oxidize cellulose group, and collagen had a significant difference. The oxidize cellulose group with the control group, collagen, had significant differences.

**Table 5:** Tukey examination.

Group Comparison		Sig.	Result
Control	Oxidize cellulose	0.000	Significant
	Collagen	0.000	Significant
Oxidize cellulose	Collagen	0.000	Significant
	Calcium alginat	0.000	Significant

Based on the Result above, oxidize sellulose has the largest average number of MSC cell clusters. To find out the difference, a Tukey test was performed with the result that oxidize cellulose was significantly different from the other groups.

## 4 Discussion

MSC cultures derived from SVF combined with scaffold and then tested for viability. The results of the study showed that oxidized cellulose had very low viability at only 19.5%. This is consistent with research from (Yen et al.) where from the study cell viability combined with oxidized cellulose was 0%. There are certain materials present in the oxidized cellulose that are toxic to cells. Collagen has a better viability test with 93.237 almost the same with the control group. It show that collagen much more better for biocompatibility compare to oxidize cellulose [7].

From the electron microscope we can see the attachment of MSC to the scaffold. Both of the scaffold has the ability to attach with MSC. Although oxidize cellulose has a better result, but in viability test oxidize cellulose has a very low degree of viability, so we choose the collagen for the scaffold of MSC from SVF because of its compatibility and attachment.

## 5 Conclusion

Collagen scaffold is ideal for MSC from SVF because of its compatibility and attachment.

## Ethical approval

This study has been approved by the Institutional Review Board of Saiful Anwar General Hospital, Malang, Indonesia.

## Sources of funding

This study does not use any sources of funding.

## Author contribution

Panji Sananta: Writing the paper, conceptualization

I Gede Made Oka Rahaditya: Data analysis and interpretation

Mohamad Ibnu Imadudin: Writing the paper

Marvin Anthony Putera: Writing the paper

Sri Andarini: Study concept

Umi Kalsum: Study concept

Edi Mustamsir: Data collection

Respati Suryanto Dradjat: Study concept and Data collection

## Registration of research studies

1. Name of the registry:
2. Unique Identifying number or registration ID:
3. Hyperlink to your specific registration (must be publicly accessible and will be checked):

## Guarantor

Panji Sananta.

## Provenance and peer review

Not commissioned, externally peer reviewed.

## Declaration of competing interest

This study does not have any conflict of interest.
